# Labral calcification plays a key role in hip pain and symptoms in femoroacetabular impingement

**DOI:** 10.1186/s13018-020-01610-z

**Published:** 2020-02-28

**Authors:** Giovanni Trisolino, Marta Favero, Dante Dallari, Enrico Tassinari, Francesco Traina, Miguel Otero, Steven R. Goldring, Mary B. Goldring, Chiara Carubbi, Roberta Ramonda, Stefano Stilli, Brunella Grigolo, Eleonora Olivotto

**Affiliations:** 1 Pediatric Orthopedic and Traumatology, IRCCS Istituto Ortopedico Rizzoli, Via G.C. Pupilli 1, 40136 Bologna, Italy; 2 Rheumatology Unit, Department of Medicine (DIMED), University Hospital of Padova, Via Giustiniani 2, 35128 Padua, Italy; 3 Reconstructive Orthopaedic Surgery Innovative Techniques - Musculoskeletal Tissue Bank, Revision surgery of hip prosthesis and development of new implants, IRCCS Istituto Ortopedico Rizzoli, Via G.C. Pupilli 1, 40136 Bologna, Italy; 4 Orthopaedic-Traumatology and Prosthetic surgery and revisions of hip and knee implants, IRCCS Istituto Ortopedico Rizzoli, Via G.C. Pupilli 1, 40136 Bologna, Italy; 5 HSS Research Institute, Hospital for Special Surgery, 535 E 70th St, New York, NY 10021 USA; 6 RAMSES Laboratory, RIT Department, IRCCS Istituto Ortopedico Rizzoli, Via di Barbiano 1/10, 40136 Bologna, Italy

**Keywords:** Femoroacetabular impingement syndrome, Arthroscopy, Synovial inflammation, Labrum, Calcification

## Abstract

**Background:**

Hip osteoarthritis (HOA) is the most common hip disorder and a major cause of disability in the adult population, with an estimated prevalence of end-stage disease and total hip replacement. Thus, the diagnosis, prevention, and treatment of the early stages of the disease in young adults are crucial to reduce the incidence of end-stage HOA.

The purpose of this study was to determine whether (1) a relationship among the inflammatory status of labrum and synovium collected from patients with femoroacetabular impingement (FAI) would exist; and (2) to investigate the associations among the histopathological features of joint tissues, the pre-operative symptoms and the post-operative outcomes after arthroscopic surgery.

**Methods:**

Joint tissues from 21 patients undergoing hip arthroscopy for FAI were collected and their histological and immunohistochemical features were correlated with clinical parameters.

**Results:**

Synovial mononuclear cell infiltration was observed in 25% of FAI patients, inversely correlated with the hip disability and osteoarthritis outcome score (HOOS) pain and function subscales and with the absolute and relative change in total HOOS. All the labral samples showed some pattern of degeneration and 67% of the samples showed calcium deposits. The total labral score was associated with increased CD68 positive cells in the synovium. The presence of labral calcifications, along with the chondral damage worsened the HOOS post-op symptoms (adjusted *R*-square = 0.76 *p* = 0.0001).

**Conclusions:**

Our study reveals a relationship between the histologic labral features, the synovial inflammation, and the cartilage condition at the time of FAI.

The presence of labral calcifications, along with the cartilage damage and the synovitis negatively affects the post-operative outcomes in patients with FAI.

## Introduction

Hip osteoarthritis (HOA) is the most common hip disorder and a major cause of disability in the adult population, with an estimated prevalence of end-stage disease and total hip replacement (THR) [1]. Thus, the diagnosis, prevention, and treatment of the early stages of the disease in young adults are crucial to reduce the incidence of end-stage HOA.

Over the last decade, evidence has mounted for a prominent etiologic role of femoroacetabular impingement (FAI) syndrome in the development of early HOA [2–4]. While FAI likely represents the most common cause of hip pain and dysfunction in young adults and an independent risk factor for early onset of HOA [5–10], relatively little is known about the events connecting the presence of FAI and the development of end-stage HOA. Moreover, although there is an initial evidence that surgical treatment of FAI may effectively improve pain and symptoms [11], there is poor information regarding the physiopathological processes that drive the HOA progression after surgical treatment of FAI [12–15].

Most of the studies regarding the physiopathology of the osteoarthritic and pre-osteoarthritic conditions have been conducted on the knee [16]. It is now well established that osteoarthritis (OA) is a whole-joint disorder involving all joint tissues [17]; synovial inflammation correlates with pain, function, and structural changes [18] and synovitis is connected with cartilage [19] and meniscal [20] pathology. In particular, meniscal damage in knee OA is a known risk factor for the incidence of OA [21].

Similar to the knee meniscus, the hip labrum is a fibrocartilaginous structure that provides joint stability and is involved in load distribution [22]. Labral deterioration or tear leads to impairment of its functions and changes the hips biomechanics. An impaired lubrication and an increased joint friction deteriorate articular cartilage and lead to OA [23]. Moreover, the labral fibrocartilage is highly innervated by nociceptors and therefore, it has been suggested as a relevant source of pain in hip and knee OA [24]. As calcium crystal deposition is common in OA menisci [25], also labral calcifications are present in patients with FAI [23]. In vitro and preclinical data demonstrate that calcium crystals can activate intra-articular proinflammatory pathways and release nociceptor stimulating substances. Thus, calcium crystal deposition may be involved in generating joint pain [26, 27].

The purposes of our study were (1) to analyze the hip joint tissues at the time of surgery for FAI, hypothesizing a relationship among the inflammatory status of labrum and synovium; and (2) to investigate the potential associations among the preoperative symptoms, the outcomes after arthroscopic surgery for FAI and the pathological features of labral tissue, in particular the presence of crystal deposition.

## Patients and methods

### Patient recruitment and clinical data collection

This observational study was conducted in accordance with the local ethical committees approval and patient consent. The main role of the ethics committee is to protect patients enrolled in the study providing good clinical care. A total of 21 patients with FAI were collected. Inclusion and exclusion criteria are listed in Table 1. At the time of hospital admission, the physician completed a report including demographic (age, sex, body mass index [BMI]) and clinical data (history of trauma, duration of symptoms). All patients completed a hip disability and osteoarthritis outcome score (HOOS), validated in Italian language (http://www.koos.nu/hoositalian.pdf) at the time of surgery. Plain anteroposterior radiographs in standing position were obtained before surgery to evaluate HOA severity using the joint space narrowing (JSN) [28] and the Kellgren-Lawrence (KL) grading system [29]. Patients were followed by visits at 1, 6, and 12 months after surgery. HOOS was re-administered at 12 months, absolute [HOOSpost - HOOSpre] and relative [(HOOSpost - HOOSpre/HOOSpre) × 100] variation of the HOOS (total and subscales) were calculated.

**Table 1:** Inclusion and exclusion criteria of patients with FAI

Inclusion criteria	Exclusion criteria
1. Age 18-60 years 2. Clinical diagnosis of symptomatic FAI and/or labral pathology (FADDIR test positive and/or FABER test positive) 3. At least one of the following patterns at the MRI or CT: 3.1. Alpha angle > 55° on radial view 3.2. L-CEA > 40° on coronal view 3.3. A-CEA > 40° on sagittal view 3.4. Cranial acetabular version < 0° on axial view 3.5. Imaging of definite labral tear 4. Planned arthroscopic surgery	1. Age < 18 years or > 60 years 2. Pregnant women, mentally disabled subjects, prisoners, inability to provide informed consensus 3. History of tumor or infection; established diagnosis of rheumatic pathology or clinical and radiographic signs of generalized OA; diabetes, obesity, neurologic disease 4. Arthroscopic surgery performed for reasons other than FAI and/or labral pathology; previous operations (including arthroscopic surgery) at the affected hip 5. Hip contracture (flexion < 90°); major hip deformities—classic hip dysplasia (L-CEA < 25°); deep acetabular socket (L-CEA > 45°); coxa valga (CDA > 135°); coxa vara (CDA < 120°); global acetabular retroversion (equatorial AV < 10°)

### Macroscopic findings and tissue sample collection

Hip arthroscopy was performed following standard approaches [30, 31], and macroscopic intra-articular pathology was graded using the Outerbridge score for chondral lesions [32], the Lage classification for labral lesion [33], and the Ilizaliturri mapping system for topographic localization [34]. Surgeons were very careful in preserving tissues and avoiding labral debridement when repair of these structures is the goal. When available, tissues of labrum and synovium from fovea (cartilage when possible) that would otherwise be discarded, were retrieved during the surgery for histological and immunohistochemical analyses. Moreover, only small specimens of synovium were collected (sometimes less than 2 mm in diameter), to avoid excessive bleeding.

### Histological analyses

Joint tissues were fixed in 4% formalin, dehydrated in 70% ethanol, and paraffin embedded. Sections of 5 m were cut using a rotatory microtome (Leika Biosytems RM2255), deparaffinized in xylene and rehydrated in ethanol for histological and immunohistochemical analyses. One experienced biologist performed all the observations and ratings.

To asses labral tissue morphology and degradation evaluating proteoglycan/collagen content, respectively, the sections were stained with hematoxylin-eosin (H&E) (Bioptica, Milano, Italy) and 0.25% Safranin-O/0.3% Fast Green (S-O-FG) (Sigma Aldrich, St Louis, MO) observed at × 10 magnification. Since the acetabular labrum has a fibrocartilaginous structure similar to the meniscus [13], we assessed the degree of histological degeneration of the labral specimens using a modified Pauli’s microscopic grading system [35]. Briefly, given that the tissue surface characteristics were analyzed only in the acetabular rim, we maintained the four grades used in the original Pauli’s grading system, but we changed the range scores for each grade, as follows: grade 1, 0-2; grade 2, 3-5; grade 3, 6-9; and grade 4, 10-12. Grade 1 represents normal tissue, grade 2 and grade 3 indicate mild and moderate degeneration respectively, and grade 4 represents severe degeneration.

To evaluate the calcification in labral samples, the sections were stained with alizarin red (AR) 1.4% pH 4.2 (Sigma A5533). Calcium crystal deposition score included four grades: 0, no deposition, from grade 1 to 3 depending on the size and number of deposits in the section [35]. For the described histological analysis, 2/3 sequential sections were scored for each case and to evaluate intra-reader reliability, 10 specimens selected randomly were scored twice with a time difference of at least 3 weeks.

For the histological assessment of synovial inflammation, the sections were stained with H&E and observed at × 20 magnification. The synovial inflammation was evaluated according to the histological synovial scoring system used by Scanzello and colleagues [20, 36, 37].

In order to validate the histological analyses on FAI patients, we also collected joint tissue samples from patients with idiopathic end-stage HOA as positive control of late stage OA, which were not included into the clinical outcome analysis.

### Immunohistochemical staining

We characterized the presence of T cells, B cells, monocytes, and plasma cells in synovial tissues using specific monoclonal antibodies against CD3 (MA1-34688, Pierce), CD20, CD68, and CD138 (M0755; M0814; M7228, DAKO). Antigen retrieval was performed using a commercial solution (S1700, DAKO). The signal was detected using a streptavidin-enzyme conjugated system (4+ Universal AP Detection kit) and the substrate Vulcan Fast Red Chromogen kit 2 (AP506US and FR805S, Biocare Medical). Isotype-matched immunoglobulins (IgG1 and IgG2a MAB002-3, R&D) were used as negative controls. Positivity of each section was semi-quantitatively evaluated according to the criteria described in Table 2. Results are expressed as the mean of positive cells per section, with 2 to 3 sequential sections analyzed for each patient. All images were captured using a Nikon Eclipse 90i microscope equipped with Nikon Imaging Software elements.

**Table 2:** Semi-quantitative evaluation of *immunohistochemical* staining

Score	0	1	2	3
CD68	Negative	≤ 10 positive cells	> 10 positive cells	< 50% positive cells
CD3-CD20	Negative	From one to half perivascular aggregate with or without focal interstitial infiltration positive	All perivascular aggregate and/or focal interstitial infiltration positive	
CD138	Negative	≤ 10 positive cells	> 10 positive cells in perivascular aggregate and/or in focal interstitial infiltration	> 10 positive cells both in perivascular aggregate and in focal interstitial infiltration

### Statistical analysis

The SPSS for Windows version 22 (IBM SPSS Inc., Chicago, IL, USA) was used for statistical analysis. Results are reported as mean ± standard deviation (SD) for continuous variables and as percentage for dichotomous and ordinal data. All data were tested for normality using the Kolmogorov-Smirnov test for continuous variables and chi-squared test for categorical variables. Given the small sample size and some irregularly distributed variables, nonparametric tests were used. Between-group differences were evaluated with Mann-Whitney *U* tests, and Spearman’s correlation coefficients were calculated. According to the criteria of Landis and Cock, coefficients of < 0.00 were considered as no agreement, 0-0.20 slight, 0.21-0.40 fair, 0.41-0.60 moderate, 0.61-0.80 substantial and, 0.81-1.00 almost perfect. Intra-rater reliability was tested using linear weighted Cohen’s kappa for ordinal variables and was found to be almost perfect for all the histological features (kappa 0.78-0.96). Univariable and multivariable analyses with general linear models were applied to adjust for lack of independence of the data. The correlations were considered significant for *p* values < 0.05.

## Results

### Patient characteristics

Clinical data of 21 patients with FAI are summarized in Table 3. Among the 21 FAI patients (13 males and 8 females; mean age 33 years with SD of 9.8; mean of the BMI of 25.05 kg/m^2^ with SD of 4.05), 6 cases had isolated CAM deformity, 3 cases isolated Pincer, and 12 mixed.

**Table 3:** Demographic and baseline clinical characteristics of patients with FAI (*N* = 21)

Characteristics	Values
Kellgren score, number:	
• 0	6
• 1	7
• 2	6
• 3	2
• 4	0
JSN (unit), mean ± SD	4.11 ± 1.75
Alpha angle, mean ± SD	77.91 ± 23.13
Acetabular retroversion, mean ± SD	12.54 ± 5.93
L-CEA, mean ± SD	39.61 ± 7.46
History of trauma, number	21
Median symptom duration, median (IQR) months	28 ± 24
Labral tear yes/no, number	18/3
Median HOOS total preoperative, median (IQR)	72.50 (77.81−66.30)
Median HOOS SPT subscale, median (IQR)	75 (81.25−64)
Median HOOS pain subscale, median (IQR)	75 (80.63−62.50)
Median HOOS ADL subscale, median (IQR)	75 (83.04−72.06)
Median HOOS Sport/Rec subscale, median (IQR)	56.25 (64.13−50)
Mean HOOS QOL subscale, median (IQR)	62.50 (75−50)

Data are shown as mean ± standard deviation (SD) or medians and interquartile ranges (IQR) depending on variable distribution. *JSN* joint space narrowing, *L-CEA* lateral-center edge angle, *HOOS* hip disability and osteoarthritis outcome score, *SPT* symptoms, *ADL* function in daily living, *Sport/Rec* function in sport and recreation, *QOL* free related quality of life, *THR* total hip replacement

Twelve cases had combined acetabular trimming and femoroplasty; 6 patients had isolated femoroplasty and 3 patients isolated acetabular trimming. Labral lesions were treated by focal debridement of the labral tear in 13 cases. Labral repair was performed in 6 cases, while in 2 patients no labral procedures were performed. Macroscopic cartilage lesions were found in 90% of the FAI patients. Isolated acetabular lesions were detected in 74% of the cases, while combined acetabular and femoral lesions were found in 26% of the cases. The Outerbridge grading of the cartilage lesions is reported in Supplementary Table 1.

During the surgery cartilage, it was not resected in patients with Outerbridge grade 0-1 and minimally removed with Outerbridge grade 2; therefore, cartilage samples for the histological analysis were collected only in 8 patients out of 21. Histological analyses showed that 7 on 8 patients showed from mild to severe cartilage degeneration and the 50% of all patients showed calcium deposits (data not shown). Histological cartilage degeneration showed substantial correlation with the CD68 immunostaining in the synovium (*r* = 0.77; *p* = 0.04) and a strong correlation with labrum calcifications (*r* = 0.89; *p* = 0.0001).

### Hip joint tissues feature at the time of surgery for FAI

#### Labral features

**Fig. 1:**
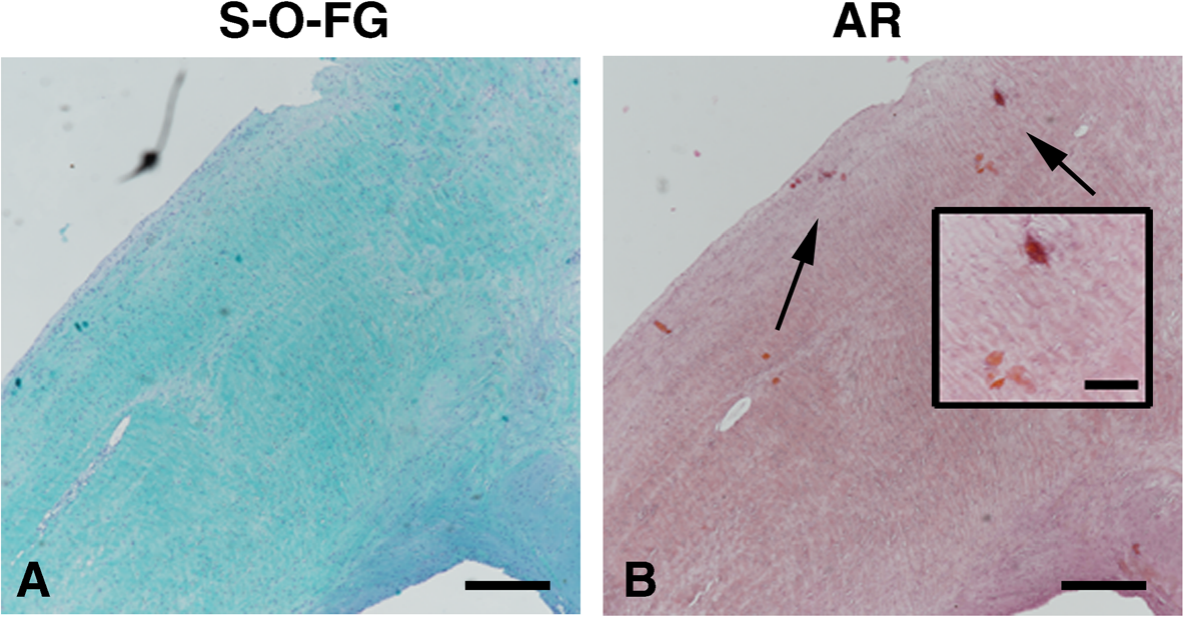
Labral tissue morphology and degradation. **a** Safranin-O-Fast green staining (S-O-FG) to access the labral degeneration from one representative FAI patient. **b** Alizarin red (AR) staining for calcium deposition in the same patients. Arrows indicate small calcium deposits also showed in the insert. Scale bar, 500 μm; insert 100 μm

Labral tears were present in 86% of the FAI patients. According to the total degeneration score, 5 patients had mild labral degeneration, 11 patients had moderate degeneration, and 2 patients severe. One representative patient is shown in Fig. 1a. The analysis of the score components is reported in Table 4.

**Table 4:** Components of the total labral degeneration score and grading of labral calcifications

	Patients with FAI (*N* = 18)					Patients with OA (*N* = 5)				
Grade	Surface	Cell.	Collagen organiz.	S-FG	Calcific.	Surface	Cell.	Collagen organiz.	S-FG	Calcific.
G 0	1	5	2	5	6					
G 1	2	6	1	10	6		5			
G 2	7	4	11	3	6	2		1	2	1
G 3	8	3	4			3		4	3	4

Data are expressed as number of patients. *G* grade, *Cell.* cellularity, *S-FG* safranin-O and fast green staining

Three components out of four (Safranin O/Fast green staining, collagen organization, and labral surface) significantly contributed to the final labral score (*r* = 0.82, *p* = 0.0001; *r* = 0.68, *p* = 0.0001; *r* = 0.54, *p* = 0.008 respectively). The collagen organization had a positive and moderate correlation with Safranin O/Fast green staining (*r* = 0.45, *p* = 0.03) and labral surface degeneration (*r* = 0.44, *p* = 0.03). Moreover, both the Safranin O/Fast green staining and the labral surface characteristics showed a positive correlation with cartilage calcification (*r* = 0.58, *p* = 0.005, and *r* = 0.61, *p* = 0.03).

Labral calcium crystal deposition was present in 67% of the FAI patients. One representative patient is shown in Fig. 1b. The increase of calcifications was moderately associated with labral degeneration (*r* = 0.42; *p* = 0.04), and both labral degeneration and calcifications increased with age (*r* = 0.61; *p* = 0.002, and *r* = 0.69; *p* = 0.0001).

The labral total score was moderately associated with the OA grading (*r* = 0.45 *p* = 0.03), the Outerbridge score at both femoral (*r* = 0.47, *p* = 0.02) and acetabular (*r* = 0.44, *p* = 0.04) levels, and substantially associated with increased CD68 positive cells in the synovium (*r* = 0.71; *p* = 0.01).

#### Synovial inflammation features

**Fig. 2:**
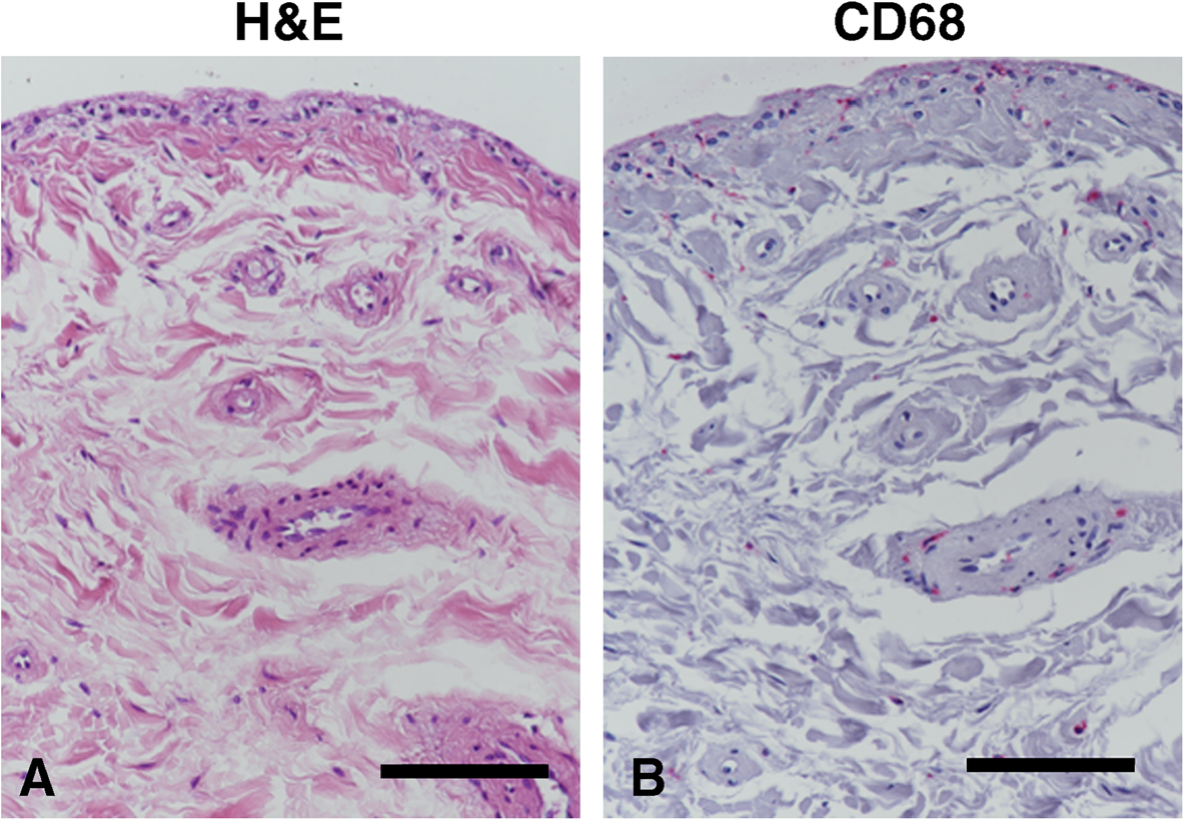
Synovial inflammation features. Synovial tissue from one representative FAI patient. **a** Hematoxylin and eosin (H&E) staining showed no monocytic cellular infiltration, mild hyperplasia, focal/perivascular fibrosis, and mild increase of vascularization. **b** CD68 immunopositive cells (red precipitate). Scale bar, 100 μm

We performed the final histological analysis on 12 patients. We discarded nine synovial samples due to the small size of the biopsies. Only the 25% of the synovial membrane samples displayed a mild perivascular mononuclear cell infiltration. Most of the synovium specimens showed signs of synovial hyperplasia, with four cases displaying 3-4 cell thick lining, five cases with more than four cells, and only three cases with normal lining. Synovial fibrosis was prevalent and only one patient had no fibrosis. Three patients showed focal/perivascular fibrosis and eight patients had widespread fibrosis. Vascularity was increased in 50% of the patients (Table 5). One representative patient is shown in Fig. 2a.

**Table 5:** Synovial histological characteristics

	Patients with FAI (*N* = 12)				Patients with OA (*N* = 4)			
Grade	PMCI (0-3)	HYP (0-2)	FIBR (0-2)	VASC (0-2)	PMCI (0-3)	HYP (0-2)	FIBR (0-2)	VASC (0-2)
G 0	9	3	1	1		2	1	
G 1	3	4	3	5		2	3	4
G 2		5	8	6	2			
G 3					2			

Data are shown as number of patients. *G* grade, *PMCI* perivascular monocyte cell infiltration, *HYP* hypertrophy, *FIBR* fibrosis, *VASC* vascularity

Among the components of the synovial score, only the mononuclear cell infiltration showed moderate or substantial association with age (*r* = 0.67; *p* = 0.004), OA grading (*r* = 0.52; *p* = 0.4), macroscopic chondral damage (*r* = 0.62; *p* = 0.01 at the acetabular side; *r* = 0.73; *p* = 0.001 at the femoral side), labral degeneration (*r* = 0.59; *p* = 0.03), and calcifications (*r* = 0.66; *p* = 0.01). Moreover, the mononuclear cell infiltration was associated with synovial hyperplasia (*r* = 0.62; *p* = 0.03).

#### Characterization of the cellular infiltration

We performed immunohistochemical analyses in 10 patients. We detected CD68 immunostaining in 8 patients: five subjects had less than 10 positive cells (grade 1); two patients had more than 10 positive cells (grade 2), and only one patient did not show any positivity. One representative patient is shown in Fig. 2b. None of these tissues showed anti-CD3, CD20, or CD138 immunopositivity.

### Correlation between clinical data and histological analysis

All patients but one completed the HOOS questionnaire at 12 months of follow up.

We observed an overall deterioration of the pre-operative hip function with older age (total HOOS *r* = − 0.50; *p* = 0.02; HOOS function *r* = − 0.49; *p* = 0.05), but a general improvement after surgery (Supplementary Figure 1). Labral calcification was inversely associated with the preoperative hip physical function (HOOS total *r* = − 0.53, *p* = 0.03; HOOS sport *r* = −0.49, *p* = 0.05), and the collagen organization (HOOS sport *r* = − 0.47; *p* = 0.05).

Post-operative hip physical function decreased with JSN (total HOOS *r* = 0.52, *p* = 0.02; absolute change total HOOS *r* = 0.45, *p* = 0.05; relative change total HOOS *r* = 0.47, *p* = 0.05) and with acetabular cartilage damage, according to the Outerbridge score (*r* = − 0.47, *p* = 0.03, *r* = − 0.52, *p* = 0.02 and *r* = − 0.48, *p* = 0.03 respectively). Moreover, the post-operative hip physical function (HOOS pain *r* = − 0.68, *p* = 0.02; HOOS function *r* = − 0.61, *p* = 0.04), and the relative change in total HOOS (*r* = −0.59, *p* = 0.05), were inversely correlated with the mononuclear cell infiltration. These latter correlations remained significant after adjustment for age and OA grading.

Finally, the post-operative clinical outcome in particular, the symptoms subscale was negatively affected by labral calcifications (*r* = − 0.51; *p* = 0.04; *n* = 17) and acetabular chondral damage (*r* = − 0.51; *p* = 0.02; *n* = 20). The association of these conditions significantly deteriorated the post-op clinical outcome (HOOS symptoms, adjusted *R*-square = 0.76 *p* = 0.0001).

## Discussion

Although there is increasing evidence that FAI and labral lesions are major risk factors for HOA onset [2, 3, 38], the chain of events that drives OA progression remains largely unknown.

We investigated the joint tissues status at the time of the surgery, focusing on labrum and synovial membrane. While the relevance of the synovial inflammation in causing the symptoms and disease progression in OA is well known, the role of the labrum has been considered mainly “mechanic” so far [22]. Labral tears may contribute not only to unfavorable joint mechanics but also to synovial inflammation and cartilage damage, increasing the risk of OA [13, 39].

Currently, there is evidence that the preservation of labral function during hip-preserving surgery improves patient outcomes and functional scores [13]. Despite this evidence, there is no consensus about which labral tears are appropriate for repair and which should be debrided. Among the factors that influence the decision to repair or debride the labral tear, the intraoperative appearance of the labrum plays the most important role [40]. In particular, the presence of labral calcifications has been indicated as a negative predictive factor affecting the quality of the surgical repair [41].

In our study, we analyzed the labrum histological features using a modified Pauli’s score [35]. We found a positive association between the total labral score and the labral calcification. Moreover, the labral calcification was also associated with increased chondral damage, synovial inflammation, and lower pre-operative HOOS and post-operative symptoms. In a recent study, Hubert et al. analyzed labral histological features of a cohort of 80 end-stage OA patients undergoing THR and found that labral calcifications were present in 100% of cases and that the total amount of calcification was inversely correlated with hip function and pain. Here we found that labral calcium crystal deposition, assessed by histology, were present in 67% of FAI patients, independent of OA radiographic grading. We also found that the presence of labral crystal calcium deposition was associated with lower pre-operative HOOS and, interestingly, with worst post-operative symptoms independent from age and radiographic severity. The same observation was previously reported [24] in end-stage OA, supporting the hypothesis that labral calcification might play a key role in hip pain and symptoms both in FAI and end-stage OA. A higher local concentration of calcium crystal deposition in the labrum could lead to increased release of nociceptor stimulating substances [26, 27] within the fibrocartilage tissue, which densely innervated [42].The presence of labral calcification is also associated with a worst labral matrix integrity and subsequent degradation of the acetabular cartilage as described by Song et al. [43],thus possibly jeopardizing the effect of the surgical repair [41].

Moreover, synovial tissue analyses revealed that most of our FAI patients had hyperplasia of the lining layer, focal, or widespread fibrosis and increased vascularity. These aspects are typical of the low-grade synovitis found in early OA cases [44]. Mononuclear cell infiltration is typical of end-stage OA [45]. Consistent with previous studies [20], we found that the presence of mononuclear cell infiltration was associated with worst clinical outcomes in patients with FAI suggesting that synovial mononuclear cell infiltration might contribute to worsen the clinical condition after hip arthroscopy [11].

Noteworthy, we found a positive association between the total labral score and CD68 expression in the synovial tissue. Abrams et al. already showed baseline levels of synovial inflammation in patients with FAI, in particular infiltration of CD68+ cell in the synovial tissue [46]. In our study, we found that patients with a severe labral degeneration had higher macrophages infiltration in the synovium.

Consistent with reports from large series of hip arthroscopies for FAI [47, 48], we found macroscopic cartilage lesions in 90% of our FAI patients, predominantly at the acetabular side. The histological analysis of the cartilage from FAI patients showed most of the features of OA patients, albeit attenuated. Cartilage degeneration was associated with the presence of macrophages (CD68+ cells) in the synovium, consistent with previous reports [46, 49]. In addition, cartilage lesions were also associated with increased calcium crystal deposition in the labrum, as previously shown for end-stage HOA [24].

The association of labral degeneration, synovial inflammation, and cartilage damage in FAI supports our hypothesis that OA, also in the early phases, affects the whole joint as an organ involving all tissues.

There are some limitations in our study related to the FAI surgery, in which the preservation of labral function is the final goal [13]. Moreover, although the Istituto Ortopedico Rizzoli is recognized by the Italian Health Ministry as Scientific Research Hospital, the patient’s care and benefit is the first aim of our surgeons in carefully preserving joint tissues during the surgical procedure.

It was not possible to collect matched tissue samples from all patients, in particular acetabular cartilage which is not resected in patients with Outerbridge grade 0-1 and minimally removed with Outerbridge grade 2. The small specimens obtained from FAI surgery, in particular synovial membrane samples (in some cases less than 1 millimeter), made the histological analysis challenging and some patients eligible for the study were therefore not included. Synovial membrane specimens were small to avoid excessive bleeding and, as for the labrum and cartilage, the synovial membrane samples were collected from discarded tissues. The sample size is limited and heterogeneous, thus, not allowing to analyze the impact of the type of surgery on the outcomes. We acknowledge that 1 year of follow up could be a very short time to assess the natural history of hip OA after surgery for FAI [50]. Nonetheless, the role of the pain and symptoms reported by the patient during the follow-up is the milestone to elucidate the results of surgery ad to propose further treatments, including early conversion to THA. A systematic review about pain, daily living activities, and returning after FAI surgery, found that the first clinically relevant improvement in hip pain was observed at 3 to 6 months after hip arthroscopy. Improvements in pain continued to postoperative 1 year [51]. Generally, we consider 1 year of follow-up, a sufficient time interval to predict if the patient could be a candidate for early revision surgery, including early conversion to THA.

Technical limitations notwithstanding, our data suggest that labral matrix integrity is important to maintain the physiological function of hip joint and therefore counteract the onset of OA pathology. Labral calcifications might play a role in pain generation and symptoms deterioration in FAI and it might be useful as a potential marker which might help orthopedics to make surgical choices. Moreover, synovial inflammation in general, and perivascular mononuclear cell infiltration in particular, might have an effect in worsening the post-operative outcome in FAI patients.

## Conclusions

Our findings provide new insights into the relationship between FAI and HOA describing (1) the features of joint tissue degeneration in patients with FAI, labral tears were present in 86% of patients and labral calcium crystal deposition in 67% of them. The 25% of the synovial membrane samples displayed a mild perivascular mononuclear cell infiltration.

In particular, (2) the presence of labral calcification associated with synovial inflammation at the time of surgery, might contribute to preoperative symptoms and to post-operative outcomes.

## Supplementary information

**Additional file 1.** Comparison between pre and post-operatively HOOS. The five panels from the left clockwise show the HOOS subscales: symptoms, pain, function, activity limitations in sport and hip related quality of life (QOL). The last panel on the bottom right shows the pre and post-operatively total HOOS. (* *p* < 0.05; ** *p* < 0.01). Scale 0-100, worst to best.

**Additional file 2: Table S1.** Acetabular and femoral chondropathy of patients with FAI at the baseline (*N* = 21).

## Data Availability

The data that support the findings of this study are available on reasonable request from the corresponding author (MF). Biological material is not available.

## Abbreviations

AR: Alizarin red
BMI: Body mass index
FAI: Femoroacetabular impingement
H&E: Hematoxylin-eosin
HOA: Hip osteoarthritis
HOOS: Hip disability and osteoarthritis outcome score
JSN: Joint space narrowing
KL: Kellgren-Lawrence
NO: Nitric oxide
SD: Standard deviation
S-O-FG: Safranin-O/Fast green
THR: Total hip replacement

## References

[1] Guillemin F, Rat AC, Mazieres B, Pouchot J, Fautrel B, Euller-Ziegler L (2011). Prevalence of symptomatic hip and knee osteoarthritis: a two-phase population-based survey. Osteoarthritis Cartilage..

[2] Vail TP (2017). CORR Insights((R)): the John Charnley award: redefining the natural history of osteoarthritis in patients with hip dysplasia and impingement. Clin Orthop Relat Res..

[3] Ganz R, Parvizi J, Beck M, Leunig M, Notzli H, Siebenrock KA (2003). Femoroacetabular impingement: a cause for osteoarthritis of the hip. Clin Orthop Relat Res..

[4] Griffin DR, Dickenson EJ, O’Donnell J, Agricola R, Awan T, Beck M (2016). The Warwick Agreement on femoroacetabular impingement syndrome (FAI syndrome): an international consensus statement. Br J Sports Med..

[5] Agricola R, Waarsing JH, Arden NK, Carr AJ, Bierma-Zeinstra SM, Thomas GE (2013). Cam impingement of the hip: a risk factor for hip osteoarthritis. Nat Rev Rheumatol..

[6] Reichenbach S, Juni P, Werlen S, Nuesch E, Pfirrmann CW, Trelle S (2010). Prevalence of cam-type deformity on hip magnetic resonance imaging in young males: a cross-sectional study. Arthritis Care Res (Hoboken)..

[7] Khanna V, Caragianis A, Diprimio G, Rakhra K, Beaule PE (2014). Incidence of hip pain in a prospective cohort of asymptomatic volunteers: is the cam deformity a risk factor for hip pain?. Am J Sports Med..

[8] Nicholls AS, Kiran A, Pollard TC, Hart DJ, Arden CP, Spector T (2011). The association between hip morphology parameters and nineteen-year risk of end-stage osteoarthritis of the hip: a nested case-control study. Arthritis Rheum..

[9] Agricola R, Heijboer MP, Bierma-Zeinstra SM, Verhaar JA, Weinans H, Waarsing JH (2013). Cam impingement causes osteoarthritis of the hip: a nationwide prospective cohort study (CHECK). Ann Rheum Dis..

[10] Clohisy JC, Dobson MA, Robison JF, Warth LC, Zheng J, Liu SS (2011). Radiographic structural abnormalities associated with premature, natural hip-joint failure. J Bone Joint Surg Am..

[11] Perets I, Chaharbakhshi EO, Shapira J, Ashberg L, Mu BH, Domb BG (2019). Hip arthroscopy for femoroacetabular impingement and labral tears in patients younger than 50 years: minimum five-year outcomes, survivorship, and risk factors for reoperations. J Am Acad Orthop Surg..

[12] Agricola R, Heijboer MP, Roze RH, Reijman M, Bierma-Zeinstra SM, Verhaar JA (2013). Pincer deformity does not lead to osteoarthritis of the hip whereas acetabular dysplasia does: acetabular coverage and development of osteoarthritis in a nationwide prospective cohort study (CHECK). Osteoarthritis Cartilage..

[13] Bsat S, Frei H, Beaule PE (2016). The acetabular labrum: a review of its function. Bone Joint J..

[14] Steppacher SD, Anwander H, Zurmuhle CA, Tannast M, Siebenrock KA (2015). Eighty percent of patients with surgical hip dislocation for femoroacetabular impingement have a good clinical result without osteoarthritis progression at 10 years. Clin Orthop Relat Res..

[15] Chandrasekaran S, Darwish N, Gui C, Lodhia P, Suarez-Ahedo C, Domb BG (2016). Outcomes of hip arthroscopy in patients with Tonnis grade-2 osteoarthritis at a mean 2-year follow-up: evaluation using a matched-pair analysis with Tonnis grade-0 and grade-1 cohorts. J Bone Joint Surg Am..

[16] Griffin DR, Dickenson EJ, Wall PDH, Realpe A, Adams A, Parsons N (2016). The feasibility of conducting a randomised controlled trial comparing arthroscopic hip surgery to conservative care for patients with femoroacetabular impingement syndrome: the FASHIoN feasibility study. J Hip Preserv Surg..

[17] Goldring MB, Otero M (2011). Inflammation in osteoarthritis. Curr Opin Rheumatol..

[18] Griffin DR, Dickenson EJ, Wall PD, Donovan JL, Foster NE, Hutchinson CE (2016). Protocol for a multicentre, parallel-arm, 12-month, randomised, controlled trial of arthroscopic surgery versus conservative care for femoroacetabular impingement syndrome (FASHIoN). BMJ Open..

[19] Scanzello CR, Goldring SR (2012). The role of synovitis in osteoarthritis pathogenesis. Bone..

[20] Scanzello CR, McKeon B, Swaim BH, DiCarlo E, Asomugha EU, Kanda V (2011). Synovial inflammation in patients undergoing arthroscopic meniscectomy: molecular characterization and relationship to symptoms. Arthritis Rheum..

[21] Edd SN, Giori NJ, Andriacchi TP (2015). The role of inflammation in the initiation of osteoarthritis after meniscal damage. J Biomech..

[22] Ferguson SJ, Bryant JT, Ganz R, Ito K (2000). The acetabular labrum seal: a poroelastic finite element model. Clin Biomech (Bristol, Avon).

[23] Domzalski ME, Synder M, Karauda A, Papierz W (2017). Histological changes of the acetabular labrum in coxarthrosis: labral degeneration and repair. Hip Int..

[24] Hubert J, Hawellek T, Moe M, Hischke S, Krause M, Rolvien T, et al. Labral calcification in end-stage osteoarthritis of the hip correlates with pain and clinical function. J Orthop Res. 2017;14. 10.1002/jor.23736 28906050

[25] Sun Y, Mauerhan DR, Honeycutt PR, Kneisl JS, Norton HJ, Zinchenko N (2010). Calcium deposition in osteoarthritic meniscus and meniscal cell culture. Arthritis Res Ther..

[26] McCarthy GM, Westfall PR, Masuda I, Christopherson PA, Cheung HS, Mitchell PG (2001). Basic calcium phosphate crystals activate human osteoarthritic synovial fibroblasts and induce matrix metalloproteinase-13 (collagenase-3) in adult porcine articular chondrocytes. Ann Rheum Dis..

[27] Morgan MP, Whelan LC, Sallis JD, McCarthy CJ, Fitzgerald DJ, McCarthy GM (2004). Basic calcium phosphate crystal-induced prostaglandin E2 production in human fibroblasts: role of cyclooxygenase 1, cyclooxygenase 2, and interleukin-1beta. Arthritis Rheum..

[28] Philippon MJ, Briggs KK, Yen YM, Kuppersmith DA (2009). Outcomes following hip arthroscopy for femoroacetabular impingement with associated chondrolabral dysfunction: minimum two-year follow-up. J Bone Joint Surg Br..

[29] Kellgren JH, Lawrence JS (1957). Radiological assessment of osteo-arthrosis. Ann Rheum Dis..

[30] Stone AV, Howse EA, Mannava S, Miller BA, Botros D, Stubbs AJ (2017). Basic hip arthroscopy: diagnostic hip arthroscopy. Arthrosc Tech..

[31] Byrd JW (2006). Hip arthroscopy. J Am Acad Orthop Surg..

[32] Outerbridge RE (1961). The etiology of chondromalacia patellae. J Bone Joint Surg Br..

[33] Lage LA, Patel JV, Villar RN (1996). The acetabular labral tear: an arthroscopic classification. Arthroscopy..

[34] Ilizaliturri VM, Byrd JW, Sampson TG, Guanche CA, Philippon MJ, Kelly BT (2008). A geographic zone method to describe intra-articular pathology in hip arthroscopy: cadaveric study and preliminary report. Arthroscopy..

[35] Pauli C, Grogan SP, Patil S, Otsuki S, Hasegawa A, Koziol J (2011). Macroscopic and histopathologic analysis of human knee menisci in aging and osteoarthritis. Osteoarthritis Cartilage..

[36] Pearle AD, Scanzello CR, George S, Mandl LA, DiCarlo EF, Peterson M (2007). Elevated high-sensitivity C-reactive protein levels are associated with local inflammatory findings in patients with osteoarthritis. Osteoarthritis Cartilage..

[37] Scanzello CR, Albert AS, DiCarlo E, Rajan KB, Kanda V, Asomugha EU (2013). The influence of synovial inflammation and hyperplasia on symptomatic outcomes up to 2 years post-operatively in patients undergoing partial meniscectomy. Osteoarthritis Cartilage..

[38] Sankar WN, Nevitt M, Parvizi J, Felson DT, Agricola R, Leunig M (2013). Femoroacetabular impingement: defining the condition and its role in the pathophysiology of osteoarthritis. J Am Acad Orthop Surg..

[39] Dhollander AA, Lambrecht S, Verdonk PC, Audenaert EA, Almqvist KF, Pattyn C (2012). First insights into human acetabular labrum cell metabolism. Osteoarthritis Cartilage..

[40] Herickhoff PK, Safran MR (2018). Surgical decision making for acetabular labral tears: an international perspective. Orthop J Sports Med..

[41] Domb BG, Hartigan DE, Perets I (2017). Decision making for labral treatment in the hip: repair versus debridement versus reconstruction. J Am Acad Orthop Surg..

[42] Alzaharani A, Bali K, Gudena R, Railton P, Ponjevic D, Matyas JR (2014). The innervation of the human acetabular labrum and hip joint: an anatomic study. BMC Musculoskelet Disord..

[43] Song Y, Ito H, Kourtis L, Safran MR, Carter DR, Giori NJ (2012). Articular cartilage friction increases in hip joints after the removal of acetabular labrum. J Biomech..

[44] Sellam J, Berenbaum F (2010). The role of synovitis in pathophysiology and clinical symptoms of osteoarthritis. Nat Rev Rheumatol..

[45] Benito MJ, Veale DJ, FitzGerald O, van den Berg WB, Bresnihan B (2005). Synovial tissue inflammation in early and late osteoarthritis. Ann Rheum Dis..

[46] Abrams GD, Luria A, Sampson J, Madding RA, Robinson WH, Safran MR (2017). Decreased synovial inflammation in atraumatic hip microinstability compared with femoroacetabular impingement. Arthroscopy..

[47] Lund B, Nielsen TG, Lind M (2017). Cartilage status in FAI patients - results from the Danish Hip Arthroscopy Registry (DHAR). SICOT J..

[48] Clohisy JC, Baca G, Beaule PE, Kim YJ, Larson CM, Millis MB (2013). Descriptive epidemiology of femoroacetabular impingement: a North American cohort of patients undergoing surgery. Am J Sports Med..

[49] Lotz M, Martel-Pelletier J, Christiansen C, Brandi ML, Bruyere O, Chapurlat R (2013). Value of biomarkers in osteoarthritis: current status and perspectives. Ann Rheum Dis..

[50] Sogbein OA, Shah A, Kay J, Memon M, Simunovic N, Belzile EL (2019). Predictors of outcomes after hip arthroscopic surgery for femoroacetabular impingement: a systematic review. Orthop J Sports Med..

[51] Kierkegaard S, Langeskov-Christensen M, Lund B, Naal FD, Mechlenburg I, Dalgas U (2017). Pain, activities of daily living and sport function at different time points after hip arthroscopy in patients with femoroacetabular impingement: a systematic review with meta-analysis. Br J Sports Med..

