# Supplementary material for: Labral calcification plays a key role in hip pain and symptoms in femoroacetabular impingement
Source: J Orthop Surg Res. 2020 Feb 28;15:86. doi: 10.1186/s13018-020-01610-z (PMC7049200; doi:10.1186/s13018-020-01610-z)
Supplement: Supplementary file 2 — Additional file 2: Table S1. Acetabular and femoral chondropathy of patients with FAI at the baseline (N = 21). [file 13018_2020_1610_MOESM2_ESM.doc]

| **Supplementary Table 1.** Acetabular and femoral chondropathy of patients with FAI at the baseline (N = 21) | |
| --- | --- |
| **Characteristics** | **Values** |
| Acetabular chondropathy, Outerbridge score, number:   - 0 - 1 - 2 - 3 - 4 | 2  4  5  5  5 |
| Femoral chondropathy, Outerbridge score, number:   - 0 - 1 - 2 - 3 - 4 | 16  1  1  2  1 |
